# Identification of Newcastle disease virus subgenotype VII.2 in wild birds in Turkey

**DOI:** 10.1186/s12917-020-02503-3

**Published:** 2020-08-08

**Authors:** Nuri Turan, Cemal Ozsemir, Aysun Yilmaz, Utku Y. Cizmecigil, Ozge Aydin, Ozge Erdogan Bamac, Aydin Gurel, Ahmet Kutukcu, Kubra Ozsemir, H. Emre Tali, Besim H. Tali, Semaha G. Yilmaz, Mehmetcan Yaramanoglu, B. Kaan Tekelioğlu, Serhat Ozsoy, Juergen A. Richt, Munir Iqbal, Huseyin Yilmaz

**Affiliations:** 1 Department of Virology, Veterinary Faculty, University of Istanbul-Cerrahpasa, Avcilar, Istanbul, Turkey; 2 Department of Pathology, Veterinary Faculty, University of Istanbul-Cerrahpasa, Avcilar, Istanbul, Turkey; 3 Department of Wild Animals and Ecology, Veterinary Faculty, University of Istanbul-Cerrahpasa, Avcilar, Istanbul, Turkey; 4 Department of Virology, Veterinary Faculty, University of Cukurova, Ceyhan, Istanbul, Turkey; 5 Department of Diagnostic Medicine and Pathobiology, College of Veterinary Medicine, Kansas State University, Manhattan, USA; 6 The Pirbright Institute, Ash Road, Pirbright, Woking, GU24 0NF UK

**Keywords:** Newcastle disease virus, Wild birds, Real time RT-PCR, Phylogenetic, Turkey

## Abstract

**Background:**

Newcastle disease viruses (NDVs) can spread across continents via migratory birds. Hence, we investigated the frequency of NDV in both non-migratory and birds migrating on the Black Sea-Mediterranean flyway, in Istanbul, Turkey. Birds were trapped using nets placed around the Kucukcekmece lake Avcilar, Istanbul, in spring seasons of 2016 and 2018. In total, 297 birds belonging to 42 different species were trapped, categorized according to species and sex, and flocked oropharyngeal swabs were collected. In addition, flocked swabs were also collected from 115 mallards caught by hunters around Edirne and from 207 birds which had been treated in the Veterinary Faculty of Istanbul university-Cerrahpasa. Tissue samples were taken from dead wild birds brought by public to Veterinary Faculty. A total of 619 flocked oropharyngeal swabs were pooled into 206 samples. RNA was extracted from swabs and tissue samples. Real-time RT-PCR prob. assay was used to detect NDV-RNA in samples.

**Results:**

There was no amplification in real time RT-PCR in samples taken from wild birds caught by traps. However, amplification of NDV-F gene was observed in oropharyngeal swabs taken from 2 waterfowls (Common Moorhen and Mallard), and in tissue samples taken from 2 little owls and 1 common kestrel. Sequencing and phylogenetic analyses of these 5 samples for NDV-F gene showed great similarity with NDV subgenotype VII.2 viruses. Analysis also showed that there is a high similarity with the F gene sequences previously reported from Turkey in 2012 and as well as the sequences from neighbouring countries Bulgaria and Georgia and geographically close country such as Pakistan. Although the strains found in this study are closely related, there is a relatively small degree of molecular divergence within 543 bp of F gene of the Turkish NDV isolate and strains detected in Israel, Pakistan, Iran, United Arab Emirates and Belgium.

**Conclusions:**

Our findings revealed the presence of subgenotype VII.2 of NDVs in wild birds in north west of Turkey and demonstrated some degree of molecular evolution when compared to the earlier NDV-VII.2 isolate in Turkey.

## Background

Newcastle disease virus (NDV) can potentially infect all species of birds and widely circulates in poultry and wild birds. According to recent classification of International Committee on Taxonomy of Viruses (ICTV), NDV (used hereafter and also known as avian paramyxovirus 1, APMV-1) belong to species *Avian orthoavulavirus 1* [1]. All NDV isolates are genetically and antigenically diverse although they belong to a single serotype. Based on genetic differences, NDV are currently classified in two major groups (class I and class II) on the basis of genetic differences. Class II viruses are known to infect a wide range of domestic and wild birds and show higher genetic and virulence variability. Although, diversity of NDV is still unfolding, they are divided into 21 genotypes named I to XXI [2].

Several pathotypes of NDV have been defined on the basis of clinical signs in domestic birds. Pathogenicity indices such as the mean death time (MDT) and the intracerebral pathogenicity index (ICPI) are usually used to classify the virus isolates into velogenic, mesogenic, and lentogenic strains. The velogenic strains (neurotrophic or viscerotropic) cause severe clinical signs and mortality in chicken. The mesogenic strains are considered moderately pathogenic and cause respiratory and neurological symptoms but with significantly low mortality. On the other hand, the lentogenic pathotypes are of low virulence, causing only mild respiratory or asymptomatic enteric disease in the affected chicken [3]. Interestingly, in some cases genetic phenotype of NDV does not translate into its pathotype potential. NDV strains isolated from pigeon and migratory ducks showed virulent cleavage site motif but were not virulent for chickens in standard pathogenicity tests [4]. Therefore, in some cases, determining virus pathogenicity has been equally important together with identification of genotypic virulence markers protein for implementation of appropriate disease control measure [3]. All domestic and wild bird species are susceptible to infection with NDV and both exchange viruses. Wild waterbirds seem to be the reservoir of avirulent strains, whereas poultry are the most likely reservoir of virulent viruses. NDV outbreaks could possibly occur due to spillover from infected wild birds [5].

The Turkish poultry industry is a significant segment of the Turkish economy and is continuously growing. The poultry in Turkey is challenged with numerous pathogens, resulting in huge economic losses every year as a consequence of these diseases. Presence of NDV in wild birds and backyard chickens threatens commercial poultry flocks [6]. Therefore, backyard chickens and wild birds need to be monitored for NDV and AIVs on a regular basis for circulating genotypes as they pose high risk of contaminating commercial poultry units. Turkey is on the flyway of the main migratory routes for wild birds which could bring an increased risk of viral diseases, such as avian influenza and Newcastle disease [6]. Istanbul and Bosphorus areas are seasonally populated by birds migrating from Eastern Europe [7]. In a recent study, 352 bird species were identified in the Istanbul area [8]. A range of bird species are reservoirs for the NDV strains with varying degree of genetic diversity [9–11]. There is likely a dynamic population for NDV which is carried along the transcontinental flyways for transmission to domestic poultry [10, 12]. However, depending on virus genotypes and pathotypes NDV rarely causes severe disease in wild birds [13]. Therefore, it is critical to monitor the virus population diversity in wild birds. Knowledge on the extent of viral burden and their genotypic and pathotypic characteristics can provide real time risk assessment about the emerging threats posed. This would allow development of appropriate disease control tools and implementation of informed disease control strategies. In this study, we therefore investigated the presence of NDV in migratory and non migratory wild birds in Turkey.

## Results

### Clinical findings in birds caught in the field and birds submitted to wildlife rehabilitation clinic

All trapped birds (297) looked clinically healthy and were not showing any clinical signs and lesions. In contrast, all birds (207) submitted to Veterinary Faculty of Istanbul exhibited a variety of clinical symptoms including exhaustion, diarrhea, emaciation and torticollis (Supplementary Figure 1). Unfortunately, one common kestrel and 2 little owls submitted to the Wildlife Rehabilitation Clinics died 2 days after clinical examination.

### Necropsy

Necropsy of the dead common kestrel and little owls revealed hemorrhagic tracheitis, proventriculitis and enteritis (Supplementary Figure 2). The walls of the intestines were thickened and there was green mucoid diarrhoea. The liver, kidneys and brain were slightly congested and enlarged. Moreover, small necrotic foci were also observed on the liver.

### Histopathological findings

**Fig. 1:**
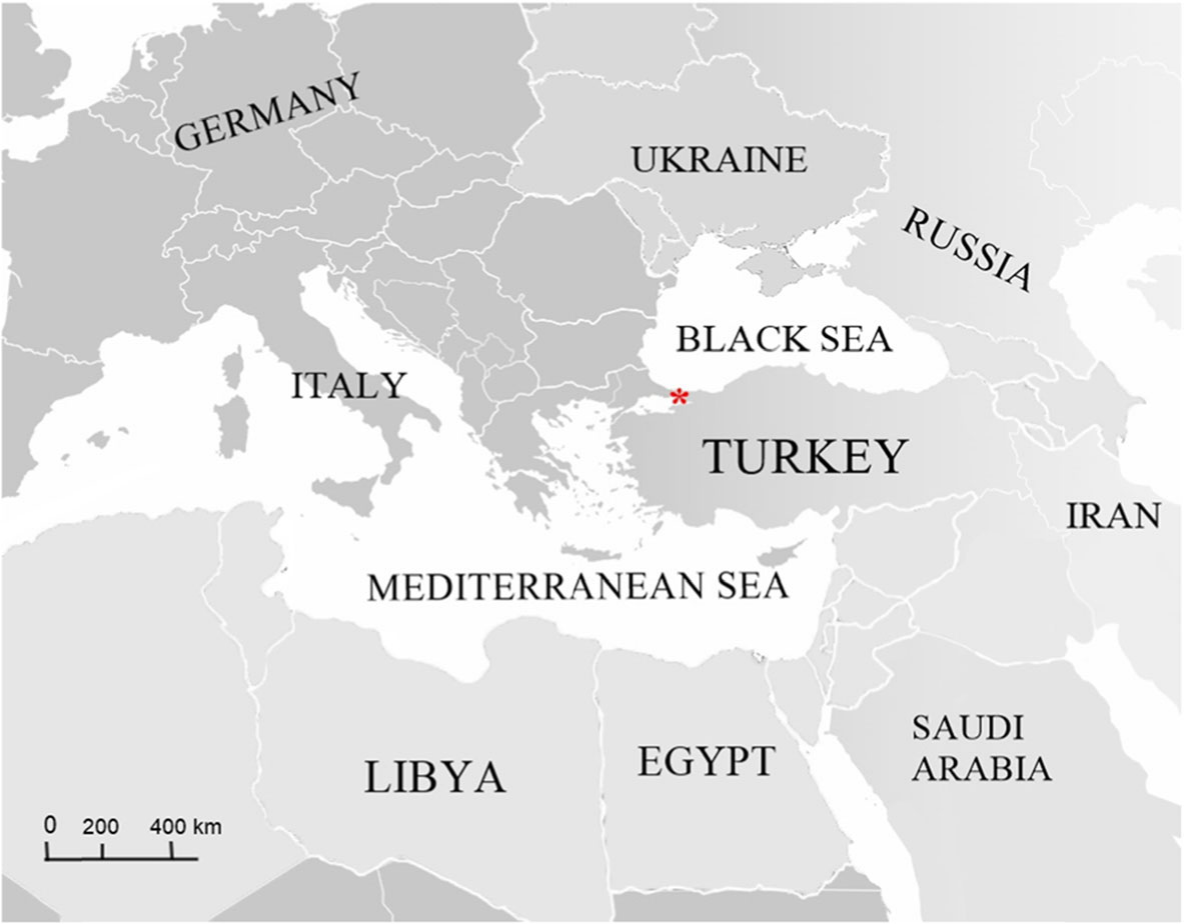
Histopathological findings in the internal organs and brain of NDV positive birds. **a**: Perivascular cuffing with mononuclear cells in the cerebellum (arrow); **b**: Necrosis in the liver (star); **c**: Foci of vacuolation in the glandular acinar tissue of pancreas; **d**: Congestion and interstitial pneumonia in lungs

Nonsuppurative meningioencephalitis was observed in all birds. The histologic changes included meningitis, mononuclear perivascular cuffing, edema, congestion and necrosis of purkinje cells. Mononuclear perivascular cuffing was the most severe lesion both in the cerebrum and cerebellum (Fig. 1-A). Necrotic hepatitis and diffuse paranchyme degeneration was prominent in the liver (Fig. 1-B). The sinusoids were dilated due to congestion. There was mild infiltration of mononuclear cells in portal regions. Hyperplasia in the bile ducts was observed. The main findings in the kidneys were congestion, mild interstitial nephritis and tubular degeneration. There was necrosis, mononuclear cell infiltration and foci of vacuolation in the glandular acinar tissue of pancreas (Fig. 1-C). Alveolar vessels were congestive in the lungs. Interstitial pneumonia (Fig. 1-D), chronic myocarditis and chronic catarrhal enteritis were observed. Hemorhages in the small intestine were prominent.

### Real time RT-PCR probe assay

There was no positive amplification signal in samples taken from birds caught by traps as well as in the negative controls. However, amplification of NDV F gene was observed in oropharyngeal swab samples taken from 2 waterfowl (Common Moorhen from the Wildlife Rehabilitation Clinic and Mallard from the hunters), and in tissue samples taken from 2 owls (*Athene noctua*) and 1 common kestrel (*Falco tinnunculus*) (Supplementary Table 1).

### Sequencing and phylogenetic analysis

All 5 samples that were found positive for NDV by real time RT-PCR were subjected to NDV F gene-specific conventional RT-PCR which resulted in 534 bp amplicons (Supplementary Figure 3) that were sequenced by Sanger sequencing. Sequences were submitted to Genbank (MK210596.1, MK210597.1, MK210598.1, MK210599.1, MK210600.1). Phylogenetic analyses revealed that all sequences clustered together with genotype VII, sub-genotype VII.2 strains with 98,72-99,08% nucleotide identity to each other (Supplementary Figure 4). Analysis showed that there is a high similarity (97,87-99,08%) with the F gene sequences previously reported from Turkey in 2012 (KP271974.1, KP271975.1, KP271976.1, KT585617.1, KT585631.1, KP271977.1) and as well as the sequences from neighbouring countries Bulgaria and Georgia (MK005972.1, KP271973.1, KP271972.1; 97,87-98,16%) and geographically close country such as Pakistan (KP271971; 97,77%).

Although the strains found in this study are closely related, there is a relatively small degree of molecular divergence within 543 bp of F gene of the Turkish NDV isolate (KT585629.1; 96,65%) and strains detected in Israel (KF792020.1, KF792021.1; 96,96%), Pakistan (JX854452, KX791186; 91,79-96,65%), Iran (MG871466.1; 96,96%), United Arab Emirates (KT995481.1; 96,81%) and Belgium (MH432252.2, 96,81%) (Supplementary Figure 4). These findings revealed that genotype VII.2 of NDV is circulating in wild birds of Eurasia Region.

## Discussion

Newcastle disease is a notifiable disease causing severe production losses and trade restriction with a significant economic impact on the poultry industry worldwide. There is increasing evidence that wild waterbirds are natural carriers of avirulent class I and class II genotypes of NDV and therefore, could play a key role in transmitting the virus in a transboundary fashion amongst regions and countries [6, 9, 11, 14] Turkey is a main flyway route for migratory birds of Europe and Asia, and thus could provide an early warning signals for circulating NDV strains between the Eurasian countries. Therefore, we determine the dominance of NDV strains prevailing in both migratory and non-migratory birds and ducks in the Marmara region of Turkey that could potentially spillover NDV strains to local commercial poultry as well as transnational dissemination risks to other countries via wild bird migration [9, 10, 12, 15]. Results of virus isolation have shown that AMPV-1 was prevalent between 0.5 and 2.5% in waterfowl including ducks [16, 17]. However, serological prevalence was reported up to 60% [17, 18]. In Turkey, 4 and 81 domestic avian ND cases were reported to OIE in 2016 and 2017, respectively. There has been no report in 2018.

In a similar study in Sanjiang natural reserve of Heilongjiang Province of China, migratory waterfowls were monitored for NDV. NDVs were isolated from waterfowls (mallard, goose, common teal and mandarin duck) [19]. In the North Sea, 543 passerine birds were investigated and the lentogenic strain of AMPV-1 was detected in 1.1% of birds [20]. In the USA, virulent strains of NDV have been found in wild birds but more frequently in pigeons, doves and double crested Cormorants. Research on NDV in wild ducks, gulls, and shorebirds found novel viral diversity, but no fusion gene sequences associated with high pathogenicity in poultry [10, 11]. However, it has been reported that most prevalent virulent genotype VII causing the endemics in Asia are co circulating into the ducks and chicken [21, 22]. Different genotypes of NDV are prevalent in both poultry and wild birds. For example, F gene of 47 NDV isolates analyzed from poultry outbreaks in Bulgaria were belong to genotypes II, IV, V and VII.1 [23]. The sub-genotype VII.1 was also found in the Middle East [24]. Later study revealed that genotype VII.1 is circulating in Bulgaria and Ukraine [15]. This sub-genotype from Bulgaria and Ukraine may have been part of a broader epizootic process in Eastern Europe rather than separate introductions from Asia or Africa. Similarly, analysis of 2 velogenic strains of NDV from ducks in China showed closer identity with genotype VII [21]. In the last few decades, genotype VI and genotype VII of NDV have been causing sporadic disease outbreaks in many countries in Asia and Europe including Denmark, Sweden, Switzerland, Austria, Hungary, Greece Germany, Belgium, Netherlands, Spain, Italy, Middle East, the Indian subcontinent and Indonesia [25]. Isolates of velogenic NDVs from domestic and synanthropic birds (pigeons, crows, and jackdaws) in Kazakhstan, Kirghizia, Ukraine, and Russia in 1993 to 2007 were sequenced and they were clustered in genotype VII comprising VII.1.1, VII.2 [6, 26].

In the past, NDV-II, VI and VII lineages were found in domestic poultry. This is the first study indicating the NDV lineage VII.2 is circulating amongst wild birds and can spread virus in and amongst countries. Lately virulent strains of NDV belonging to genotype VII have been causing severe diseases outbreaks in poultry in many neighboring countries of Turkey. Genotype VII.1.1 has been isolated from Bulgaria and Ukraine between 2002 and 2013 [23, 25]. Iran has reported poultry outbreaks with genotype VII.1.1 and VII.2 [27, 28]. These studies conclude that genotype VII is a dominant strain in poultry and wild migratory birds and gradually undergoing adaptive changes, retaining fitness to survive in both immune and naturally exposed birds.

Our study validates these findings that all sequences clustered together with genotype VII, subgenotype VII.2 strains with 98,72-99,08% nucleotide identity to each other. There is a high similarity (97,87-99,08%) with the F gene sequences previously reported from Turkey in 2012 [6] and as well as the sequences from neighbouring countries Bulgaria and Georgia (97,87-98,16%) and geographically close country such as Pakistan (97,77%) suggesting that this genotype remains endemic. However our data indicate that recent Turkish strains of this study showed some degree of molecular evolution when compared to the earlier Turkish NDV isolate [6] (96,65%) and strains detected in Israel (96,96%), Pakistan (91,79-96,65%), Iran (96,96%), United Arab Emirates (96,81%) and Belgium (96,81%). This indicates, multiple variants of genotype VII.2 are co-circulating in birds, indicating possible intercontinental spill over. The presence of a certain NDV strains in a neighbor countries poses a risk to Turkish poultry.

The currently used modified live viruses, LaSota and Hitchner-B1vaccine strains are clustering on a different branch of the phylogenetic tree than the NDV isolates obtained in this study. As suggested recently by Dimitrov and others [29], efficacy of the above mentioned modified live vaccines against the presently circulating NDV strains and vaccine application failures needs to be taken into consideration. Therefore, new vaccination strategies may be required for NDV in chickens in the field.

## Conclusions

Results of this study revealed that NDV-VII.2 is circulating amongst wild birds in Turkey as in other countries. Therefore, continued surveillance of NDV in both migratory birds and poultry is critical for assessment of genetic traits of these viruses. This can only be achieved through establishment of stronger national and international collaborations performing regional surveillance and improving disease control strategies.

## Methods

### Description of the wild bird trapping area

**Fig. 2:**
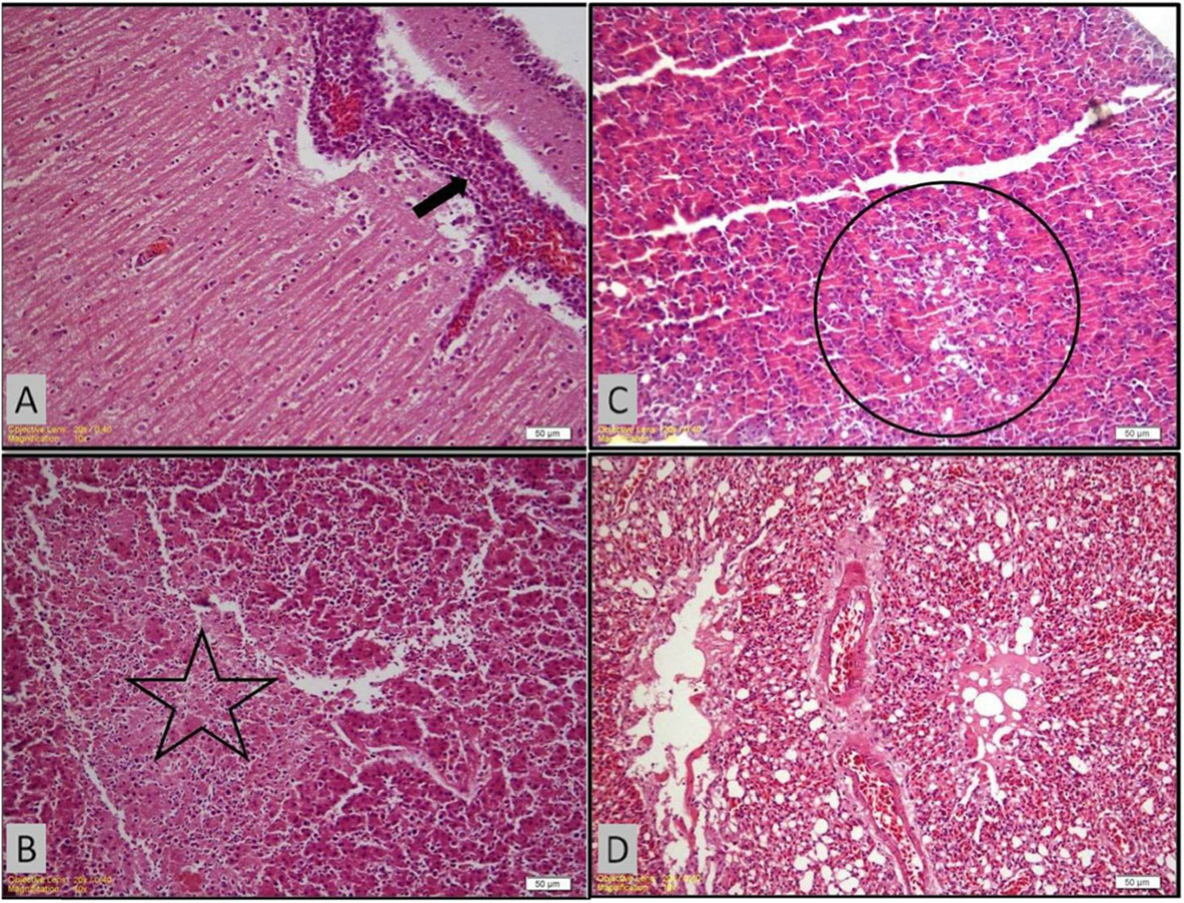
Area of bird traps around Kucukcekmece lake in Avcilar, Istanbul (Star; 40°59ˈN, 28°44ˈE). Bird traps were placed in this area and left for observation. Trapped birds were sampled and released. The figure was created in Adobe Creative Cloud program (v4.3.0.256)

In the present study, field work was performed on the Black Sea-Mediterranean flyway of wild birds in the Marmara region of Turkey. Traps were placed around the Kucukcekmece Lake in Avcilar, Istanbul, in order to catch migratory and non-migratory wild birds (Fig. 2). The lake contains brakish water and is about 16 km^2^ in size, surrounded by villages, agricultural areas and forests with oaks, ash trees, shrubbery, and turpentine trees [8].

### Bird traps, wild bird population and collection of samples

Mist nets were placed around Kucukcekmece Lake (Fig. 2) in spring 2016 and 2018 for 50 days. The targeted bird population was migratory and non-migratory wild birds. The traps were nylon nets, black in colour and 4 × 12 meter in diameter. They were left open from sunrise to sunset and checked hourly according to instructions established by the South East European Network (SEEN) for researchers [30]. For sample collection, approval and permission were taken from the Ethics Committee of the Istanbul University-Cerrahpasa (Ethics No: 2016/39). Moreover, a legal permission to do field studies was taken from the Ministry of Forestry of Turkey and local legislation rules were strictly followed as well as international guidelines. The birds caught were categorized according to species and sex (in species having sexual dimorphism) as described previously [31]. A total of 297 birds belonging to 42 species were trapped (Supplementary Table 1). To avoid duplicate sampling, they were ringed under the rules and the licenses given by the General Directorate of Nature Conservation and National Parks of the Ministry of Agriculture and Forestry in Turkey.

Dry oropharyngeal swabs were collected from trapped birds using a commercially available nylon flocked swab (Copan Flock Technologies Srl, Brescia, Italy; 503CS01). In addition, dry oropharyngeal swabs were also taken from 115 mallards (*Anas platyrhynchos*) which were caught by hunters in Edirne area of Turkey. Birds were immediately released after taking oropharyngeal swabs. All swab samples were immediately transported to the laboratory under cold storage (4–8 °C).

### Wild birds submitted to wildlife rehabilitation clinic

A total of 207 Wild birds (31 species) were brought by citizens to Wildlife Rehabilitation Clinic at the Veterinary Faculty of Istanbul University. Birds species were recorded, clinical examination was peroformed and oropharyngeal swabs (Copan Flock Technologies Srl, Brescia, Italy; 503CS01) were taken. These birds were kept for the rehabilitation in an isolated room till they got cured and released to the appropriate environment. Some of the birds were taken back by the same citizens who brought the birds to the Wildlife Rehabilitation Clinic.

In addition, 18 tissues (3 brain, 3 trachea, 3 lung, 3 liver, 3 pancreas and 3 intestine) were taken during necropsies from dead birds (owls, common kestrels) submitted to Istanbul Veterinary Faculty of Istanbul (originally examined in the Wildlife Rehabilitation Clinic) and wild birds submitted to Ceyhan Veterinary Faculty.

### Histopathology

Tissue samples (brain, lung, liver, pancreas and intestine) from the dead birds were analysed histopathologically. For this, samples were fixed in 10% neutral buffered formalin, embedded in paraffin blocks, cut into 4–5 μm sections, stained with hematoxylin and eosin (HE), and blindly examined. Histopathological analyses were carried out by two certified veterinary pathologists in the Department of Pathology.

### RNA extraction and reverse transcription

Tubes containing oropharyngeal swabs were vortexed individually after adding 500 μl of nuclease free water. A total of 619 oropharyngeal swabs were pooled into 206 samples (619:3) pools by mixing 100 μl of each swab sample to make a 300 μl pool, then 140 μl were taken for RNA extraction using Qiagen® RNeasy Mini Kit (Qiagen®, Valencia, CA, USA) as per manufacturer instructions. Tissue samples taken from dead birds were homogenized using the ribolyser (Hybaid, UK) and RNA was extracted using RNeasy mini kit (Qiagen®, Valencia, CA, USA) as per manufacturer instructions.

The amount of RNA in the eluted samples (50 μl) was measured using a NanoDrop spectrophotometer (NanoDrop 1000c, Thermo Scientific, Waltham, USA). RNA (about 100 ng) was subjected to reverse transcription for generation of cDNA using reverse transcription kit (Applied Biosystems, ThermoFisher Scientific, Carlsbad, CA) as described by the manufacturer.

### Real time RT-PCR probe assay for NDV

All samples were analyzed by real time RT-PCR assays for the matrix gene of NDV. Primers and probes used to detect NDV-RNA were described previously [32]. During optimization of the assay, optimal amplification signals were obtained when F and R primers were used in a concentration of 10 pmol/μl with 4 μl cDNA when using the positive controls. An optimized real-time RT-PCR reaction consisted of a 25 μl mixture containing of 12.5 μl Maxima/ROX qPCR Master Mix (Thermo Scientific, Catalog No: K0232), 1.25 μl forward primer (10 pMol / μl), 1.25 μl reverse primer (10 pMol / μl), 0.4 μl probe (10 pMol / μl), 2.5 μl cDNA and 7.1 μl nuclease free water. The mixture was placed in a thermal cycler (Stratagene Mx3000P, Agilent Technologies) and the polymerase activated by incubation at 95 °C for 10 min. Cycling conditions were 95 °C for 15 s, 52 °C for 30 s and 72 °C for 10 s over 40 cycles. For all PCR reactions, nuclease-free water was used as negative control of the PCR assay in place of the template and NDV specific RNA (LA Sota Live vaccine strain) as positive control.

### RT-PCR for sequencing partial NDV-F gene

Primers used for sequencing parts of the NDV-F gene were designed based on a previous study [33]. Samples found to be positive for NDV by real time RT-PCR were subjected to RT-PCR as described previously [33]. An optimised RT-PCR reaction consisted of a 25 μl mixture containing of 12.5 μl Maxima/ROX qPCR Master Mix (Thermo Scientific, Catalog No:K0232), 2 μl forward primer (20 pMol / μl), 2 μl reverse primer (20 pMol / μl), 0.5 μl MgCl_2_ (25 nM), 1 μl 2% DMSO, 5 μl nuclease free water and 2 μl cDNA. The mixture was placed in a thermal cycler (Stratagene Mx3000P, Agilent Technologies) and the polymerase activated by incubation at 95 °C for 10 min. Cycling conditions were 94 °C for 30 s, 55 °C for 30 s and 72 °C for 30 s over 30 cycles. 72 °C for 5 min of final extension step was added at the end of the reaction. For all PCR reactions, nuclease-free water was used as negative control in place of template as well as NDV specific RNA as positive control. After the PCR, the presence of the 534 bp product for NDV-F gene was confirmed by agarose gel (1.5%) electrophoresis. Products obtained by RT-PCR using the primers specific for the partial NDV-F gene were sequenced (Sanger sequencing) by a commercial company (MedSanTek, Istanbul, Turkey).

### Phylogenetic analysis

The nucleotide sequences of the NDV F gene were processed by BioEdit program and a BLAST search was performed to determine the most related NDV F gene sequences in GenBank. Final dataset was created with the addition of the reference sequence set for NDV obtained from previously published data [2] and aligned with ClustalW multiple alignment method. Multiple alignments of the NDV F gene region sequences were made using the MEGA-X software. Phylogenetic analyses were carried out using the criterion of maximum likelihood methods using Kimura 2-parameter model with gamma distribution with invariant sites (K2 + G + I) and 1000 replicates of bootstrap by Tamura and others [34]. The partial NDV F gene sequences obtained in this study were submitted to GenBank (MK210596, MK210597, MK210598, MK210599, MK210600).

## Supplementary information

**Additional file 1: Table S1.** Species of birds, number of PCR positives and number of samples collected in this study. *Non-passerine species. **Wild birds submitted to the Wildlife Clinic of the Veterinary Faculty of Istanbul.

**Additional file 2: Figure S1.** Picture of wild birds which were found to be positive for NDV-RNA by real time RT-PCR. A common kestrel (A) and 2 little owls (B). **Figure S2.** Necropsy findings of the dead common kestrel. Hemorrhages and necrosis seen in the proventriculus. **Figure S3.** Sequencing PCR for NDV. A: 100 bp Marker; B: Positive control; D: Negative control; C and E: Positive samples; Other wells: Negative samples.

**Additional file 3: Figure S4.** Maximum-likelihood phylogenetic tree based on partial NDV-F gene (534 bp) sequences. Strain classification has been performed using the reference sequences submitted to GenBank. Black dots indicate strains detected in this study.

## Data Availability

The data generated and/or analyzed during this study are available from the corresponding author upon request. The sequences of F gene of NDV detected in this study was submitted to GenBank (Accession numbers: MK210596, MK210597, MK210598, MK210599, MK210600).

## Abbreviations

ND: Newcastle disease
NDV: Newcastle disease virus
HN: Hemagglutinin-neuraminidase
F: Fusion
OIE: Office Internationale des Epizootics
SEEN: South East European Network
