# Supplementary material for: Identification of Newcastle disease virus subgenotype VII.2 in wild birds in Turkey
Source: BMC Vet Res. 2020 Aug 8;16:277. doi: 10.1186/s12917-020-02503-3 (PMC7414739; doi:10.1186/s12917-020-02503-3)
Supplement: Supplementary file 1 — Additional file 1: Table S1. Species of birds, number of PCR positives and number of samples collected in this study. *Non-passerine species. **Wild birds submitted to the Wildlife Clinic of the Veterinary Faculty of Istanbul. [file 12917_2020_2503_MOESM1_ESM.docx]

**Supplementary Document Table 1.**

| **No** | **Local name** | **English name** | **Species** | **Number of PCR positives** | **Number of birds** |
| --- | --- | --- | --- | --- | --- |
| **1**** | Bahri* | Great Crested Grebe | *Podiceps cristatus* | 0 | 1 |
| **2**** | Küçük balaban* | Little Bittern | *Ixobrychus minutus* | 0 | 4 |
| **3**** | Küçük ak balıkçıl* | Little Egret | *Egretta garzetta* | 0 | 1 |
| **4**** | Gri balıkçıl* | Grey Heron | *Ardea cinerea* | 0 | 1 |
| **5**** | Erguvani balıkçıl* | Purple Heron | *Ardea purpurea* | 0 | 1 |
| **6**** | Leylek* | White Stork | *Ciconia ciconia* | 0 | 2 |
| **7**** | Kuğu* | Mute Swan | *Cyngus olor* | 0 | 6 |
| **8**** | Yeşilbaş* | Mallard | *Anas platyrhynchos* | 1 | 115 |
| **9**** | Arı şahini* | European Honey Buzzard | *Pernis apivornus* | 0 | 2 |
| **10**** | Saz delicesi* | Western Marsh Harrier | *Circus aeruginosus* | 0 | 3 |
| **11**** | Gökçe delice* | Hen Harrier | *Circus cyaneus* | 0 | 1 |
| **12**** | Çakırkuşu* | Northern Goshawk | *Accipiter gentilis* | 0 | 2 |
| **13**** | Atmaca * | Eurasian Sparrowhawk | *Accipiter nisus* | 0 | 5 |
| **14**** | Şahin* | Common Buzzard | *Buteo buteo* | 0 | 7 |
| **15**** | Kızıl Şahin* | Long-legged Buzzard | *Buteo rufinus* | 0 | 1 |
| **16**** | Küçük orman kartalı* | Lesser Spotted Eagle | *Clanga pomarina* | 0 | 1 |
| **17**** | Kerkenez* | Common Kestrel | *Falco tinnunculus* | 1 | 3 |
| **18**** | Ala doğan* | Red-footed Falcon | *Falco vespertinus* | 0 | 1 |
| **19**** | Gök doğan* | Peregrine Falcon | *Falco peregrinus* | 0 | 1 |
| **20**** | Su klavuzu* | Water Rail | *Rallus aquaticus* | 0 | 1 |
| **21**** | Bataklık suyelvesi* | Little Crake | *Porzana parva* | 0 | 1 |
| **22**** | Bıldırcın klavuzu* | Corn Crake | *Crex crex* | 0 | 7 |
| **23**** | Su tavuğu* | Common Moorhen | *Gallinula chloropus* | 1 | 2 |
| **24**** | Sakarmeke* | Eurasian Coot | *Fulica atra* | 0 | 20 |
| **25**** | Çulluk* | Eurasian Woodcock | *Scolopax rusticola* | 0 | 4 |
| **26**** | Karabaş martı* | Black-headed Gull | *Chroicocephalus ridibundus* | 0 | 2 |
| **27**** | Gümüş martı* | Yellow-legged Gull | *Larus michahellis* | 0 | 7 |
| **28**** | Peçeli baykuş* | Western Barn Owl | *Tyto alba* | 0 | 2 |
| **29** | İshakkuşu* | Eurasian Scops Owl | *Otus scops* | 0 | 10 |
| **30**** | Kukumav* | Little Owl | *Athene noctua* | 2 | 4 |
| **31**** | Alaca baykuş* | Tawny Owl | *Strix aluca* | 0 | 2 |
| **32** | Çobanaldatan* | European Nightjar | *Caprimulgus europaeus* | 0 | 3 |
| **33** | Arıkuşu* | European Bee-eater | *Merops apiaster* | 0 | 2 |
| **34** | Yeşil ağaçkakan* | European Green Woodpecker | *Picus viridis* | 0 | 1 |
| **35** | Alaca ağaçkakan* | Syrian Woodpecker | *Dendrocopos syriacus* | 0 | 1 |
| **36** | Kır kırlangıcı | Barn Swallow | *Hirundo rustica* | 0 | 2 |
| **37** | Ağaç incirkuşu | Tree Pipit | *Anthus trivialis* | 0 | 4 |
| **38** | Akyanaklı arapbülbülü | White-eared Bulbul | *Pycnonatus leucotis* | 0 | 1 |
| **39** | Çitkuşu | Eurasian Wren | *Troglodytes troglodytes* | 0 | 3 |
| **40** | Kızılgerdan | European Robin | *Erithacus rubecula* | 0 | 6 |
| **41** | Benekli bülbül | Thrush Nightingale | *Luscinia lusciana* | 0 | 6 |
| **42** | Bülbül | Common Nightingale | *Luscinia megarhynchos* | 0 | 16 |
| **43** | Kızılkuyruk | Common Redstart | *Phoenicurus phoenicurus* | 0 | 4 |
| **44** | Karatavuk | Common Blackbird | *Turdus merula* | 0 | 8 |
| **45** | Öter ardıç | Song Thrush | *Turdus philomelos* | 0 | 4 |
| **46** | Kamış bülbülü | Cetti’s Warbler | *Cettia cetti* | 0 | 1 |
| **47** | Bataklık kamışçını | Savi’s Warbler | *Locustella lusciniodies* | 0 | 1 |
| **48** | Kındıra kamışçını | Sedge Warbler | *Acrocephalus schoenobaenus* | 0 | 1 |
| **49** | Saz kamışçını | Eurasian Reed Warbler | *Acrocephalus scirpaceus* | 0 | 5 |
| **50** | Ak mukallit | Eastern Olivaceous Warbler | *Iduna pallida* | 0 | 5 |
| **51** | Maskeli ötleğen | Sardinian Warbler | *Sylvia melanocephala* | 0 | 1 |
| **52** | Akgözlü ötleğen | Eastern Orpean Warbler | *Sylvia crassirostris* | 0 | 2 |
| **53** | Çizgili ötleğen | Barred Warbler | *Sylvia nisoria* | 0 | 3 |
| **54** | Küçük akgerdanlı ötleğen | Lesser Whitethroat | *Sylvia curruca* | 0 | 18 |
| **55** | Akgerdanlı ötleğen | Common Whitethroat | *Syvia communis* | 0 | 9 |
| **56** | Boz ötleğen | Garden Warbler | *Sylvia borin* | 0 | 1 |
| **57** | Karabaşlı ötleğen | Eurasian Blackcap | *Sylvia atricapilla* | 0 | 58 |
| **58** | Sarıkaşlı çıvgın | Yellow-browed Warbler | *Phylloscopus inornatus* | 0 | 1 |
| **59** | Orman çıvgını | Wood Warbler | *Phylloscopus sibilatrix* | 0 | 1 |
| **60** | Çıvgın | Common Chiffchaff | *Phylloscopus collybita* | 0 | 20 |
| **61** | Söğüt bülbülü | Willow Warbler | *Phylloscopus trochilus* | 0 | 15 |
| **62** | Benekli sinekkapan | Spotted Flycatcher | *Muscicapa striata* | 0 | 5 |
| **63** | Küçük sinekkapan | Red-breasted Flycatcher | *Ficedula parva* | 0 | 21 |
| **64** | Alaca sinekkapan | Semicollared Flycatcher | *Ficedula semitorquata* | 0 | 4 |
| **65** | Halkalı sinekkapan | Collared Flycatcher | *Ficedula albicollis* | 0 | 25 |
| **66** | Kara sinekkapan | European Pied Flycatcher | *Ficedula hypoleuca* | 0 | 16 |
| **67** | Büyük baştankara | Great Tit | *Parus major* | 0 | 3 |
| **68** | Sarıasma | Eurasian Golden Oriole | *Oriolus oriolus* | 0 | 1 |
| **69** | Kızılsırtlı örümcek kuşu | Red-backed Shrike | *Lanius collurio* | 0 | 3 |
| **70** | Saksağan | Eurasian Magpie | *Pica pica* | 0 | 2 |
| **71**** | Kuzgun* | Northen Raven | *Corvus corax* | 0 | 1 |
| **72** | Serçe | House Sparrow | *Passer domesticus* | 0 | 4 |
| **73** | İspinoz | Common Chaffinch | *Fringilla coeleps* | 0 | 1 |
| **Total** |  |  |  | 5 | 509 |
