# Supplementary material for: Identification of Newcastle disease virus subgenotype VII.2 in wild birds in Turkey
Source: BMC Vet Res. 2020 Aug 8;16:277. doi: 10.1186/s12917-020-02503-3 (PMC7414739; doi:10.1186/s12917-020-02503-3)
Supplement: Supplementary file 2 — Additional file 2: Figure S1. Picture of wild birds which were found to be positive for NDV-RNA by real time RT-PCR. A common kestrel (A) and 2 little owls (B). Figure S2. Necropsy findings of the dead common kestrel. Hemorrhages and necrosis seen in the proventriculus. Figure S3. Sequencing PCR for NDV. A: 100 bp Marker; B: Positive control; D: Negative control; C and E: Positive samples; Other wells: Negative samples. [file 12917_2020_2503_MOESM2_ESM.docx]

**Supplementary Figure 1:** Picture of wild birds which were found to be positive for NDV-RNA by real time RT-PCR. A common kestrel (A) and 2 little owls (B).


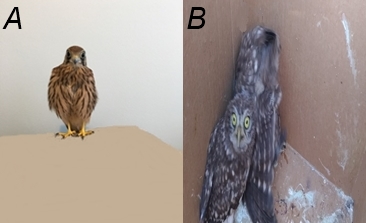


**Supplementary Figure 2:** Necropsy findings of the dead common kestrel. Hemorrhages and necrosis seen in the proventriculus.


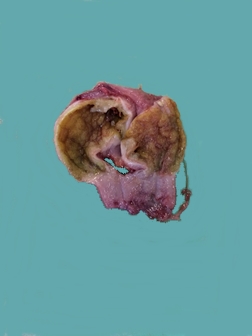


**Supplementary Figure 3:** Sequencing PCR for NDV. A: 100 bp Marker; B: Positive control; D: Negative control; C and E: Positive samples; Other wells: Negative samples.

**
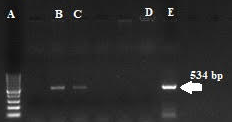
**
