# Supplementary material for: Identification of Newcastle disease virus subgenotype VII.2 in wild birds in Turkey
Source: BMC Vet Res. 2020 Aug 8;16:277. doi: 10.1186/s12917-020-02503-3 (PMC7414739; doi:10.1186/s12917-020-02503-3)
Supplement: Supplementary file 3 — Additional file 3: Figure S4. Maximum-likelihood phylogenetic tree based on partial NDV-F gene (534 bp) sequences. Strain classification has been performed using the reference sequences submitted to GenBank. Black dots indicate strains detected in this study. [file 12917_2020_2503_MOESM3_ESM.pdf]

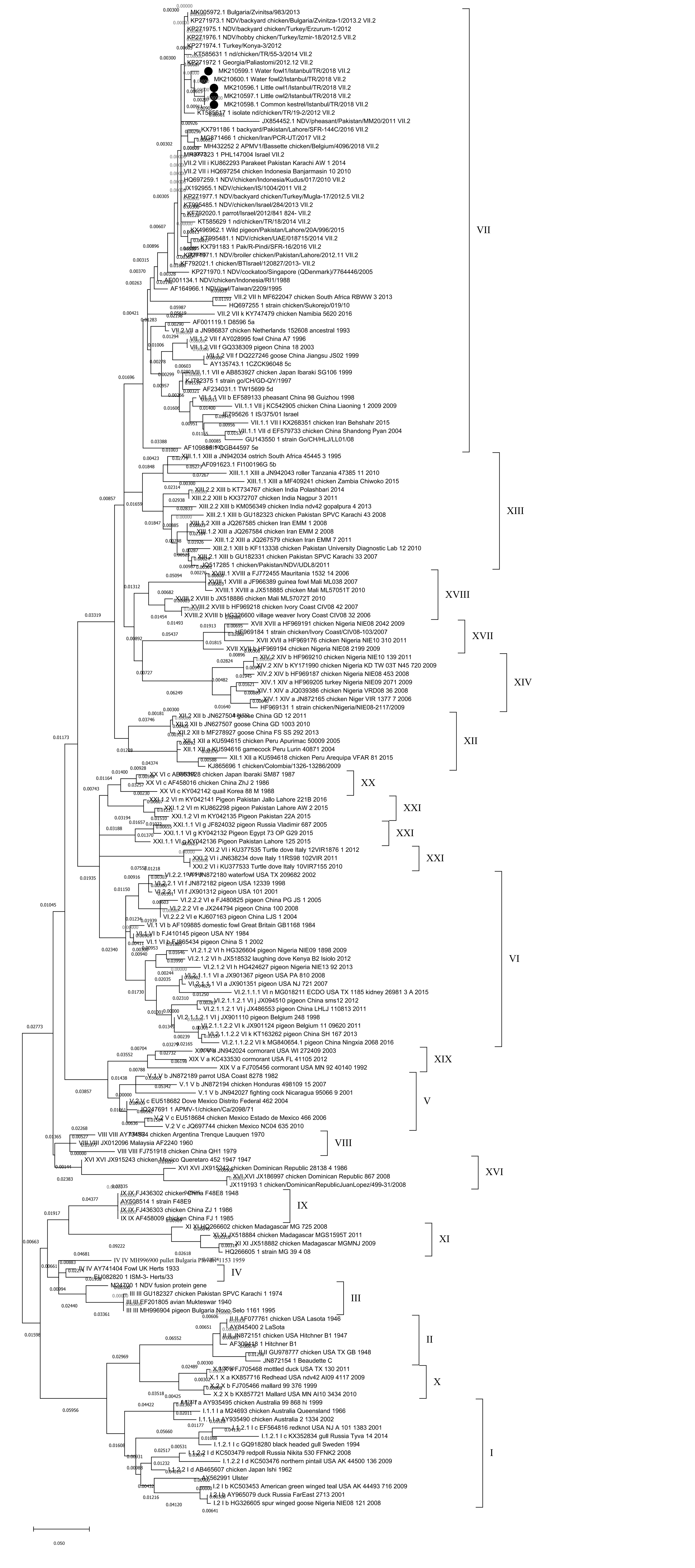

MK005972.1 Bulgaria/Zvinitsa/983/2013
KP271973.1 NDV/backyard chicken/Bulgaria/Zvinitza-1/2013.2 VII.2
KP271975.1 NDV/backyard chicken/Turkey/Erzurum-1/2012
KP271976.1 NDV/hobby chicken/Turkey/Izmir-18/2012.5 VII.2
KP271974.1 Turkey/Konya-3/2012
KT585631 1 nd/chicken/TR/55-3/2014 VII.2
KP271972 1 Georgia/Paliastomi/2012.12 VII.2
MK210599.1 Water fowl1/Istanbul/TR/2018 VII.2
MK210600.1 Water fowl2/Istanbul/TR/2018 VII.2
MK210596.1 Little owl1/Istanbul/TR/2018 VII.2
MK210597.1 Little owl2/Istanbul/TR/2018 VII.2
MK210598.1 Common kestrel/Istanbul/TR/2018 VII.2
KT585617 1 isolate nd/chicken/TR/19-2/2012 VII.2
JX854452.1 NDV/pheasant/Pakistan/MM20/2011 VII.2
KX791186 1 backyard/Pakistan/Lahore/SFR-144C/2016 VII.2
MG871466 1 chicken/Iran/PCR-UT/2017 VII.2
MH432252 2 APMV1/Bassette chicken/Belgium/4096/2018 VII.2
MH377323 1 PHL147004 Israel VII.2
VII.2 VII i KU862293 Parakeet Pakistan Karachi AW 1 2014
VII.2 VII i HQ697254 chicken Indonesia Banjarmasin 10 2010
HQ697259.1 NDV/chicken/Indonesia/Kudus/017/2010 VII.2
JX192955.1 NDV/chicken/IS/1004/2011 VII.2
KP271977.1 NDV/backyard chicken/Turkey/Mugla-17/2012.5 VII.2
KT965485.1 NDV/chicken/Israel/284/2013 VII.2
KF792020.1 parrot/Israel/2012/841 824- VII.2
KT585629 1 nd/chicken/TR/18/2014 VII.2
KX496962.1 Wild pigeon/Pakistan/Lahore/20A/996/2015
KT995481.1 NDV/chicken/UAE/018715/2014 VII.2
KX791183 1 Pak/R-Pindi/SFR-16/2016 VII.2
KP271971.1 NDV/broiler chicken/Pakistan/Lahore/2012.11 VII.2
KF792021.1 chicken/BTIsrael/120827/2013- VII.2
KP271970.1 NDV/cockatoo/Singapore (QDenmark)/7764446/2005
AF001134.1 NDV/chicken/Indonesia/RI1/1988
AF164966.1 NDV/owl/Taiwan/2209/1995
VII.2 VII h MF622047 chicken South Africa RBWW 3 2013
HQ697255 1 strain chicken/Sukorejo/019/10
VII.2 VII k KY747479 chicken Namibia 5620 2016
AF001119.1 D8596 5a
VII.2 VII a JN986837 chicken Netherlands 152608 ancestral 1993
VII.1.2 VII f AY028995 fowl China A7 1996
VII.1.2 VII f GQ338309 pigeon China 18 2003
VII.1.2 VII f DQ227246 goose China Jiangsu JS02 1999
AY135743.1 1CZCK96048 5c
VII.1.1 VII e AB853927 chicken Japan Ibaraki SG106 1999
KJ782375 1 strain go/CH/GD-QY/1997
AF234031.1 TW15699 5d
VII.1.1 VII b EF589133 pheasant China 98 Guizhou 1998
VII.1.1 VII j KC542905 chicken China Liaoning 1 2009 2009
JF795626 1 IS/375/01 Israel
VII.1.1 VII l KX268351 chicken Iran Behshahr 2015
VII.1.1 VII d EF579733 chicken China Shandong Pyan 2004
GU143550 1 strain Go/CH/HLJ/LL01/08
AF109886.1 QGB44597 5e
VII
XIII.1.1 XIII a JN942034 ostrich South Africa 45445 3 1995
AF091623.1 FI100196G 5b
XIII.1.1 XIII a JN942043 roller Tanzania 47385 11 2010
XIII.1.1 XIII a MF409241 chicken Zambia Chiwoko 2015
XIII.2.2 XIII b KT734767 chicken India Polashbari 2014
XIII.2.2 XIII b KX372707 chicken India Nagpur 3 2011
XIII.2.2 XIII b KM056349 chicken India ndv42 gopalpura 4 2013
XIII.2.1 XIII b GU182323 chicken Pakistan SPVC Karachi 43 2008
XIII.1.2 XIII a JQ267585 chicken Iran EMM 1 2008
XIII.1.2 XIII a JQ267584 chicken Iran EMM 2 2008
XIII.1.2 XIII a JQ267579 chicken Iran EMM 7 2011
XIII.2.1 XIII b KF113338 chicken Pakistan University Diagnostic Lab 12 2010
XIII.2.1 XIII b GU182331 chicken Pakistan SPVC Karachi 33 2007
JQ517285 1 chicken/Pakistan/NDV/UDL8/2011
XIII
XVIII.1 XVIII a FJ772455 Mauritania 1532 14 2006
XVIII.1 XVIII a JF966389 guinea fowl Mali ML038 2007
XVIII.1 XVIII a JX518885 chicken Mali ML57051T 2010
XVIII.2 XVIII b JX518886 chicken Mali ML57072T 2010
XVIII.2 XVIII b HF969218 chicken Ivory Coast CIV08 42 2007
XVIII.2 XVIII b HG326600 village weaver Ivory Coast CIV08 32 2006
XVIII
XVII XVII a HF969191 chicken Nigeria NIE08 2042 2009
HF969184 1 strain chicken/Ivory Coast/CIV08-103/2007
XVII XVII a HF969176 chicken Nigeria NIE10 310 2011
XVII XVII b HF969194 chicken Nigeria NIE08 2199 2009
XVII
XIV.2 XIV b HF969210 chicken Nigeria NIE10 139 2011
XIV.2 XIV b KY171990 chicken Nigeria KD TW 03T N45 720 2009
XIV.2 XIV b HF969187 chicken Nigeria NIE08 453 2008
XIV.1 XIV a HF969205 turkey Nigeria NIE09 2071 2009
XIV.1 XIV a JQ039386 chicken Nigeria VRD08 36 2008
XIV.1 XIV a JN872165 chicken Niger VIR 1377 7 2006
HF969131 1 strain chicken/Nigeria/NIE08-2117/2009
XIV
XII.2 XII b JN627504 goose China GD 12 2011
XII.2 XII b JN627507 goose China GD 1003 2010
XII.2 XII b MF278927 goose China FS SS 292 2013
XII.1 XII a KU594615 chicken Peru Apurimac 50009 2005
XII.1 XII a KU594616 gamecock Peru Lurin 40871 2004
XII.1 XII a KU594618 chicken Peru Arequipa VFAR 81 2015
KJ865696 1 chicken/Colombia/1326-13286/2009
XII
XX VI c AB853928 chicken Japan Ibaraki SM87 1987
XX VI c AF458016 chicken China ZhJ 2 1986
XX VI c KY042142 quail Korea 88 M 1988
XX
XXI.1.2 VI m KY042141 Pigeon Pakistan Jallo Lahore 221B 2016
XXI.1.2 VI m KU862298 pigeon Pakistan Lahore AW 2 2015
XXI.1.2 VI m KY042135 Pigeon Pakistan 22A 2015
XXI.1.1 VI g JF824032 pigeon Russia Vladimir 687 2005
XXI.1.1 VI g KY042132 Pigeon Egypt 73 OP G29 2015
XXI.1.1 VI g KY042136 Pigeon Pakistan Lahore 125 2015
XXI
XXI
XXI.2 VI i KU377535 Turtle dove Italy 12VIR1876 1 2012
XXI.2 VI i JN638234 dove Italy 11RS98 102VIR 2011
XXI.2 VI i KU377533 Turtle dove Italy 10VIR7155 2010
XXI
VI.2.2.1 VI f JN872180 waterfowl USA TX 209682 2002
VI.2.2.1 VI f JN872182 pigeon USA 12339 1998
VI.2.2.1 VI f JX901312 pigeon USA 101 2001
VI.2.2.2 VI e FJ480825 pigeon China PG JS 1 2005
VI.2.2.2 VI e JX244794 pigeon China 100 2008
VI.2.2.2 VI e KJ607163 pigeon China LJS 1 2004
VI.1 VI b AF109885 domestic fowl Great Britain GB1168 1984
VI.1 VI b FJ410145 pigeon USA NY 1984
VI.1 VI b FJ865434 pigeon China S 1 2002
VI.2.1.2 VI h HG326604 pigeon Nigeria NIE09 1898 2009
VI.2.1.2 VI h JX518532 laughing dove Kenya B2 Isiolo 2012
VI.2.1.2 VI h HG424627 pigeon Nigeria NIE13 92 2013
VI.2.1.1.1 VI a JX901367 pigeon USA PA 810 2008
VI.2.1.1.1 VI a JX901351 pigeon USA NJ 721 2007
VI.2.1.1.1 VI n MG018211 ECDO USA TX 1185 kidney 26981 3 A 2015
VI.2.1.1.2.1 VI j JX094510 pigeon China sms12 2012
VI.2.1.1.2.1 VI j JX486553 pigeon China LHLJ 110813 2011
VI.2.1.1.2.1 VI j JX901110 pigeon Belgium 248 1998
VI.2.1.1.2.2 VI k JX901124 pigeon Belgium 11 09620 2011
VI.2.1.1.2.2 VI k KT163262 pigeon China SH 167 2013
VI.2.1.1.2.2 VI k MG840654.1 pigeon China Ningxia 2068 2016
VI
XIX V a JN942024 cormorant USA FL 41105 2012
XIX V a KC433530 cormorant USA FL 41105 2012
XIX V a FJ705456 cormorant USA MN 92 40140 1992
XIX
V.1 V b JN872189 parrot USA Coast 8278 1982
V.1 V b JN872194 chicken Honduras 498109 15 2007
V.1 V b JN942027 fighting cock Nicaragua 95066 9 2001
V.2 V c EU518682 Dove Mexico Distrito Federal 462 2004
JQ247691 1 APMV-1/chicken/Ca/2098/71
V.2 V c EU518684 chicken Mexico Estado de Mexico 466 2006
V.2 V c JQ697744 chicken Mexico NC04 635 2010
V
VIII VIII AY734534 chicken Argentina Trenque Lauquen 1970
VIII VIII JX012096 Malaysia AF2240 1960
VIII VIII FJ751918 chicken China QH1 1979
VIII
XVI XVI JX915243 chicken Mexico Queretaro 452 1947 1947
XVI XVI JX915242 chicken Dominican Republic 28138 4 1986
XVI XVI JX186997 chicken Dominican Republic 867 2008
JX119193 1 chicken/DominicanRepublicJuanLopez/499-31/2008
XVI
IX IX FJ436302 chicken China F48E8 1948
AY508514 1 strain F48E9
IX IX FJ436303 chicken China ZJ 1 1986
IX IX AF458009 chicken China FJ 1 1985
IX
XI XI HQ266602 chicken Madagascar MG 725 2008
XI XI JX518884 chicken Madagascar MGS1595T 2011
XI XI JX518882 chicken Madagascar MGMNJ 2009
HQ266605 1 strain MG 39 4 08
XI
IV IV MH996900 pullet Bulgaria Plovdiv 1153 1959
IV IV AY741404 Fowl UK Herts 1933
EU082820 1 ISM-3- Herts/33
IV
M24700 1 NDV fusion protein gene
III III GU182327 chicken Pakistan SPVC Karachi 1 1974
III III EF201805 avian Mukteswar 1940
III III MH996904 pigeon Bulgaria Novo Selo 1161 1995
III
II II AF077761 chicken USA Lasota 1946
AY845400 2 LaSota
II II JN872151 chicken USA Hitchner B1 1947
AF309418 1 Hitchner B1
II II GU978777 chicken USA TX GB 1948
JN872154 1 Beaudette C
II
X.1 X a FJ705468 mottled duck USA TX 130 2011
X.1 X a KX857716 Redhead USA ndv42 AI09 4117 2009
X.2 X b FJ705466 mallard 99 376 1999
X.2 X b KX857721 Mallard USA MN AI10 3434 2010
X
I.1.1 I a AY935495 chicken Australia 99 868 hi 1999
I.1.1 I a M24693 chicken Australia Queensland 1966
I.1.1 I a AY935490 chicken Australia 2 1334 2002
I.1.2.1 I c EF564816 redknot USA NJ A 101 1383 2001
I.1.2.1 I c KX352834 gull Russia Tyva 14 2014
I.1.2.1 I c GQ918280 black headed gull Sweden 1994
I.1.2.2 I d KC503479 redpoll Russia Nikita 530 FFNK2 2008
I.1.2.2 I d KC503476 northern pintail USA AK 44500 136 2009
I.1.2.2 I d AB465607 chicken Japan Ishi 1962
AY562991 Ulster
I.2 I b KC503453 American green winged teal USA AK 44493 716 2009
I.2 I b AY965079 duck Russia FarEast 2713 2001
I.2 I b HG326605 spur winged goose Nigeria NIE08 121 2008
I
0.050
